# NOX signaling in molecular cardiovascular mechanisms involved in the blood pressure homeostasis

**DOI:** 10.3389/fphys.2015.00194

**Published:** 2015-07-07

**Authors:** Mariarosaria Santillo, Antonio Colantuoni, Paolo Mondola, Bruna Guida, Simona Damiano

**Affiliations:** Dipartimento di Medicina Clinica e Chirurgia, Università di Napoli “Federico II”Naples, Italy

**Keywords:** NOX, blood pressure, reactive oxygen species, redox signaling, cardiomyocytes, endothelial cells, vascular smooth muscle cells, Ang II signaling

## Abstract

Blood pressure homeostasis is maintained by several mechanisms regulating cardiac output, vascular resistances, and blood volume. At cellular levels, reactive oxygen species (ROS) signaling is involved in multiple molecular mechanisms controlling blood pressure. Among ROS producing systems, NADPH oxidases (NOXs), expressed in different cells of the cardiovascular system, are the most important enzymes clearly linked to the development of hypertension. NOXs exert a central role in cardiac mechanosensing, endothelium-dependent relaxation, and Angiotensin-II (Ang-II) redox signaling regulating vascular tone. The central role of NOXs in redox-dependent cardiovascular cell functions renders these enzymes a promising pharmacological target for the treatment of cardiovascular diseases, including hypertension. The aim of the present review is to focus on the physiological role of the cardiovascular NOX-generating ROS in the molecular and cellular mechanisms affecting blood pressure.

## Blood pressure regulation

Blood pressure is regulated by a dynamic equilibrium of different complex mechanisms (Opie, 2004; Raven and Chapleau, 2014). The main factor determining the systemic blood pressure is the blood arterial volume that, in turn, depends on the cardiac output and vascular resistances. In addition to the nervous and chemical factors, the cardiac output, is affected by mechanical factors ensuring the adjustment of cardiac output to the venous return and afterload. On the other hand, vascular resistance depends in part on the characteristics of the blood (viscosity) and on the diameter of the vascular lumen. Smooth muscle cell layer of the resistance arteries may contract or relax resulting in a parallel increase or decrease of blood pressure, respectively. Several mechanisms regulate vascular tone. Adrenergic sympathetic fibers exert a vasoconstrictory effect through the activation of alfa1-adrenergic receptors of the vascular smooth muscle cells (VSMCs). In addition, paracrine, hormonal and mechanical mechanisms contribute to the fine regulation of vascular tone modulating blood pressure.

## NOX isoforms

### NOXs structure and activation

NOX enzymes are membrane NADPH oxidases with the unique role of producing superoxide anions by one electron reduction of oxygen using NAD(P)H as electron donor (Bedard and Krause, 2007).

NADPH oxidase, first discovered in phagocytes (Segal and Jones, 1978) is a multicomponent complex comprising two integral membrane proteins, the catalytic subunit gp91phox (now referred to as NOX2) and p22phox, and the cytosolic components p47phox, p67phox, p40phox, and Rac1 or 2 (Dinauer et al., 1987; Knaus et al., 1991; Babior et al., 2002). Upon stimulation, cytosolic subunits translocate to the membrane activating the enzyme.

**Figure 1:**
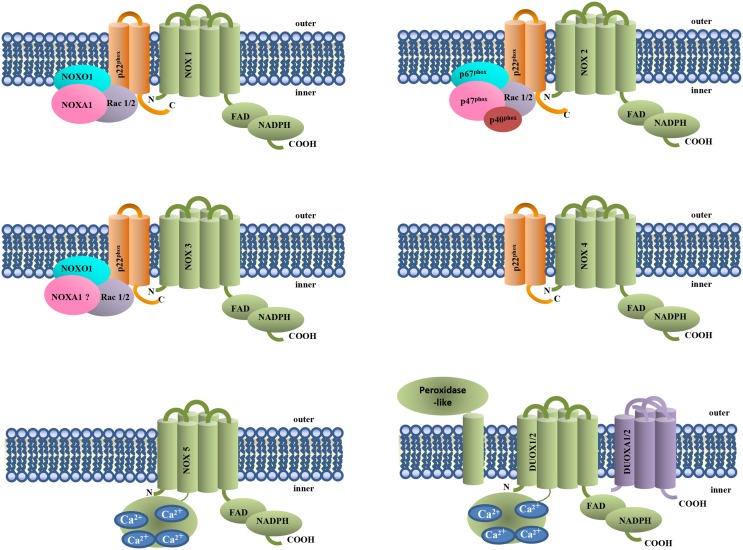
**NOX isoforms and regulatory subunits**. NOX1-4 are associated to the membrane subunit p22phox. NOX4, NOX5, and DUOX1 and 2 do not require cytosolic subunits for their activity. NOX5 and DUOX1 and 2 activation requires Ca^2+^ binding to their EF-hand domains.

Up to now, in mammalian, seven different NOX genes (NOX1 to 5 and DUOX1 and 2) have been identified (Lambeth, 2004) (Figure 1). Like NOX2, also NOX1, NOX3, and NOX4 are associated with p22phox, but the mechanisms of activation are different. NOX1 is activated by membrane translocation of the cytosolic subunits NOXO1, NOXA1, and Rac 1or 2, while NOX3 requires NOXO1 but is still uncertain the role of the other cytosolic subunits. NOX4, NOX5, DUOX 1, and 2 activity is not modulated by cytosolic subunits. DUOX 1 and 2 terminate at N-terminus with an extracellular peroxidase-homology domain (PHD) (Donko et al., 2005) and together with NOX5, are modulated by calcium. NOX4 and DUOX1 and 2 produce hydrogen peroxide instead of superoxide anion (Martin et al., 2006).

### NOXs functions

ROS have been for long time considered as toxic byproducts of the chemical utilization of oxygen within the cells and oxidative stress has been linked to the pathogenesis of many disorders (Sies, 1991; Cuda et al., 2002; Sabbatini et al., 2006; Ruocco et al., 2007; Bertoni et al., 2011). It was the discovery of NOX enzymes, that deliberately produce ROS, to highlight the physiological role of ROS (Santillo et al., 2001; Schieber and Chandel, 2014). Many redox-dependent biological processes are controlled through the fine regulation of ROS-producing systems and antioxidant enzymes like glutathione peroxidase, catalase and superoxide dismutases (Mondola et al., 2004; Santillo et al., 2007; Secondo et al., 2008; Cassano et al., 2010; Damiano et al., 2013; Terrazzano et al., 2014).

Multiple physiological functions have been so far attributed to NOX enzymes. DUOX1 and 2 enzymes, first discovered in thyroid with a role in thyroid hormones synthesis (De Deken et al., 2000), are involved also in the innate immunity and cell signaling (Harper et al., 2005; der Vliet, 2008; Bae et al., 2010). NOXs-generated ROS modulate several physiological processes such as cell growth and differentiation or mucin expression and secretion (Ris-Stalpers, 2006; Chan et al., 2009; Damiano et al., 2015).

NOXs are activated by growth factor receptors such as platelet-derived growth factor receptor (Svegliati et al., 2005; Baroni et al., 2006; Gabrielli et al., 2008; Damiano et al., 2012), epidermal growth factor receptor (Damiano et al., 2015) cholinergic receptors (Seru et al., 2004) and many others (Petry et al., 2010). NOX-dependent ROS, in turn, regulate phosphorylation levels of multiple proteins modulated by redox-sensitive kinases and/or protein phosphatase (Brown and Griendling, 2009).

An interesting feature of NOXs biology is that ROS generated by NOXs can act downstream on other ROS generating systems (Lassègue et al., 2012). For example, in endothelial cells, oxidative stress associated with oscillatory shear stress is mediated by NOX-dependent xanthine oxidase activation (McNally et al., 2003). Moreover, there are evidences of a cross talk among NOX homologs. As an example, platelet-derived growth factor induces DUOX1-2 levels in human neuroblastoma cells through NOX2-derived ROS (Damiano et al., 2012). Therefore, the activation of NOXs results in a ROS-induced ROS release that can lead to oxidative stress and associated diseases. In human coronary arteries a correlation between NOXs mRNA expression and severity of atherosclerotic lesions has been shown (Sorescu et al., 2002).

## NOXs in the cardiovascular system

NOXs are expressed in cardiomyocytes and in all cells of the vascular wall including endothelial cells (ECs), vascular smooth muscle cells (VSMCs), and adventitial fibroblasts.

### Endothelial NOXs

ECs express NOX1, NOX2, NOX4, and NOX5 (Jones et al., 1996; Damico et al., 2012). They regulate cell differentiation, proliferation, migration, angiogenesis and vascular tone. NOX2 has been clearly linked to the reduced bioavailability of endothelium-derived NO (Görlach et al., 2000); NOX1 mediates cell growth, while NOX4 growth suppression, probably depending on their different subcellular localization or agonist stimulation. However, the specific role of the different NOX homologs should be better clarified. NOXs in ECs are in part localized at plasma membrane producing extracellular superoxide with a paracrine function (Barbacanne et al., 2000), and in part preassembled in intracellular compartments also with a perinuclear distribution (Li and Shah, 2002). Their functions are crucial for of ECs activation by different stimuli that rely on redox sensitive signaling molecules. Among the main targets of NOX-derived ROS in ECs there are the transcriptional factors NF-kB, Activated protein-1, hypoxia-inducible factor-1 or p53 that regulate gene expression. Endothelial NOXs also activates signaling cascades acting on protein kinases, (p38 and c-Jun N-terminal kinase, protein kinase B and Src), and/or protein phosphatases, including protein tyrosine phosphatase (Damico et al., 2012).

### NOXs in VSMCs

NOX2 is the main isoform of VSMCs of resistance arteries, while its expression is very low in VSMCs of large vessels (Lassegue and Clempus, 2003). NOX1 and NOX4 are expressed in VSMCs of large arteries with distinct subcellular localization and functions. NOX1, less abundant respect to NOX4, is localized at caveolae and mediates cell proliferation, while NOX4, that induces cell differentiation, is mainly localized at focal adhesions, the sites of tyrosine kinase signaling (Hilenski et al., 2004). Human aortic VSMCs also express NOX5 (Jay et al., 2008). In addition to NOX2, resistance arteries express NOX4, while the expression of NOX1 has not been so far clearly demonstrated.

### NOXs in cardiomyocytes

NOX2 and NOX4 are the most abundantly expressed isoforms in cardiomyocytes. NOX2 is localized in plasma membrane and is modulated by stretch or Ang II (Byrne et al., 2003) (see NOXs in the cardiomyocyte force development). NOX4, involved in cell differentiation (Murray et al., 2013), is constitutively active and is mainly localized in intracellular compartments (Zhang et al., 2010).

## NOXs in ang II signaling

Ang II, the major bioactive peptide of the Renin-Angiotensin System (RAS), is involved in many vascular processes including vasoconstriction, fibrosis, hypertrophy, inflammation, and aging (Mehta and Griendling, 2007). These effects are mediated by the interaction of Ang II with AT-1 receptor, while AT-2 receptor activation results in opposite vasodilatatory and antiproliferative effects. Vascular NOXs, including NOX1, NOX2, NOX4, and NOX5, are all regulated by Ang II (Nguyen Dinh Cat et al., 2013) that promotes an increase in blood pressure. The Ang II effects are mainly mediated by NOX-derived ROS signaling (Montezano et al., 2014). In addition to a direct effect on VSMCs, Ang II strengthens the sympathetic vasoconstriction increasing the synthesis and the release of the neurotransmitter at adrenergic varicosities. It has been also demonstrated that NOX-dependent ROS, in turn, activate AT-1 receptor thereby potentiating cell signaling with an auto amplificatory effect (Nishida et al., 2011).

Ang II induces activation of the enzymatic activity and increases expression of NOXs both in cultured VSMCs and intact arteries (Virdis et al., 2004). ROS produced thereby, activate multiple redox sensitive molecule including mitogen-activated protein, non-receptor tyrosine kinases, protein tyrosine phosphatases, calcium channels, and redox-sensitive transcription factors. In addition, Ang II activates tyrosine kinase receptors by transactivation (Cruzado et al., 2005; Li et al., 2010). Activation of these signaling pathways modulates different cellular processes in VSMCs, including contraction that relies on an increase of intracellular calcium levels and on the activation of the RhoA/Rho kinase pathway (Touyz and Schiffrin, 2000; Touyz et al., 2005), leading to an increase of myosin light chain phosphorylation. AT-1 receptor signaling activated by Ang II have an hypertrophic and fibrotic effect on the cardiac cells, mediated in part by endothelin-1 (ET-1) release (Weng et al., 2015). Moreover, Ang II redox signaling in cardiomyocytes is also involved in the *Anre*p effect (see NOXs in the cardiomyocyte force development). For the role of NOX-dependent Ang II signaling in the endothelium see NOXs in the endothelium-dependent relaxation.

## NOXs in the endothelium-dependent relaxation

NOX signaling have a role in endothelium dependent vasorelaxation that is mainly mediated by nitric oxide (NO) generated by endothelial nitric oxide synthase (eNOS). The liposolubile NO diffuses across the membranes reaching VSMCs, where it increases cGMP levels by activating the soluble guanylate cyclase; the subsequent activation of cGMP-dependent kinases leads to a decrease of intracellular calcium levels and relaxation. Superoxide anions produced by NOXs react with NO to produce peroxynitrite (Beckman et al., 1990; Görlach et al., 2000), leading to reduced bioavailability of NO and vasoconstriction. In addition, it has been shown that in aortas of mice with deoxycorticosterone acetate–salt (DOCA-salt) hypertension, ROS produced by NOXs oxidize the eNOS cofactor tetrahydrobiopterin, leading to the uncoupling of eNOS that produces superoxide rather than NO (Landmesser et al., 2003). This mechanism can cooperate with the scavenging effects of superoxide ions, leading to a reduced NO levels and impairment of endothelium-dependent vasorelaxation. This evidence has been also demonstrated *in vivo* using NOX1 overexpressing mice. In these animals subjected to Ang II induced hypertension, endothelium-dependent relaxation was impaired and bioavailable NO was markedly decreased (Dikalova et al., 2010). In ECs, Ang II activates all NOXs expressed in these cells including NOX5. Ang II-dependent extracellular signaling regulated kinase1/2 activation that mediate growth and inflammation, relies on NOX5 superoxide production. However, unlike the other NOX homologs, NOX5 overexpression increases eNOS activity even if, due to its NO antagonistic action, the overall effect is an impairing of endothelium-dependent relaxation, similarly to the other NOX homologs (Zhang et al., 2008).

## NOXs in the cardiomyocyte force development

NOX-generating ROS contribute to the positive inotropic response to mechanical stretch in cardiomyocytes. Physiological stretch triggers a microtubule-mediated activation of NOX2 localized at t-tubule membranes. This mechanism referred by Prosser et al. (2011) as X-ROS signaling, produces ROS that can diffuse across the membrane to oxidize the RyR2 Ca^2+^ release channels, located at junctional sarcoplasmic reticulum (J-SR) close to NOX2. Then, ryanodine receptors-2 activation leads to an increase of local cytosolic Ca^2+^ concentration and force development (Prosser et al., 2011, 2013; Sag et al., 2013). It has been also demonstrated that a cycling cardiomyocyte stretch *in vitro* results in an increase of ROS levels correlated with the amplitude and the frequency of stretch (Prosser et al., 2013). This mechanism could be of relevant physiological significance *in vivo* during the normal cyclic stretching and shortening of cardiomyocytes at each heartbeat, where the Ca^2+^ spark can be dynamically modulated by ROS in dependence of pre-load and heart frequency.

NOX2 is also involved in the slow enhanced increase in intracellular Ca^2+^ concentration and myocardial contractility due to mechanical stretch, known as *Anrep* effect. This slow response follows within 1–2 min an increase of the afterload reaching a maximum after 10–15 min. In this case, NOX2-derived ROS mediates Ang II dependent ET-1 release. In cardiomyocytes, Ang II released by mechanical stretch (Sadoshima et al., 1993) induces NOX2 activating auto-AT1 receptors, and induces ET-1 release (Ito et al., 1993). ET-1 signaling activates Na^+^/H^+^ exchanger-1 (Akram et al., 2006), that results in an increase of intracellular Na^+^, inhibition of Na^+^/Ca^2+^ exchanger, increase of cytosolic calcium concentration and contraction.

The role of NOXs in cardiomyocytes are not limited to mechanosensing. ROS produced by NOXs and by other sources such as mitochondria, are able to modulate different kinases phosphorylating proteins involved in calcium signaling; sarco/endoplasmic reticulum Ca^2+^-ATPase, plasma membrane Ca^2+^ ATPase, L-Type Ca^2+^ channels and Na_*v*_ are some examples of molecular target of ROS leading to modulation of intracellular Ca^2+^ levels linked to myocyte contractility (Sag et al., 2013).

## NOXs in hypertension

The role of ROS in hypertension has been well documented (Lee and Griendling, 2008; Sirker et al., 2011).

Numerous studies using ROS scavengers or more specific NOX inhibitors, were aimed at evaluating the role of NOXs in the elevation of blood pressure in hypertensive animals (Lassègue et al., 2012). More recently, the involvement of different NOX homologs and of their regulatory subunits in the pathogenesis of hypertension have been investigated using transgenic knockout and overexpressing animal models. These studies evidenced that NOX homologs exert different effects on hypertension.

NOX2 elevation is correlated with hypertension. Indeed, in transgenic mice with endothelial-specific overexpression of NOX2, Ang II causes a greater increase in ROS production and attenuated acetylcholine-induced vasorelaxation compared to wild-type (Murdoch et al., 2011). On the other hand, NOX2 knockout mice show baseline and Ang II-induced blood pressure values significantly lower than that of wild-type animals, even if the increase in blood pressure related to baseline is comparable in the two strains (Wang et al., 2001). Similar results were obtained in p47phox knockout animals in which the lack of Ang II hypertensive response was associated with a strong decrease of Ang II-dependent superoxide production in ECs and VSMCs (Landmesser et al., 2002). Unlike NOX2, cardiomyocyte-targeted NOX4 have protective effects facilitating cardiac adaptation to chronic cardiac pressure overload (Zhang et al., 2010; Schröder et al., 2012). Also endothelial NOX4 exerts beneficial effects. Transgenic mice with endothelium-targeted NOX4 overexpression show enhanced acetylcholine- or histamine-induced vasodilatation than wild-type animals. It is noteworthy to remind that NOX4 produces hydrogen peroxide instead of superoxide so preserving NO bioavailability (Ray et al., 2011). Moreover, when in hypertension or atherosclerosis eNOS was uncoupled to produce superoxide rather than NO (Landmesser et al., 2003), endothelial NOX4-derived H_2_O_2_ could mediate compensatory relaxation acting as an endothelium-derived hyperpolarizing factor (Yada et al., 2003).

Human studies aimed at linking NOX dysfunction with hypertension, have shown that some polymorphisms in the gene encoding p22 phox and affecting enzymatic activity, are associated with hypertension (Zalba et al., 2005). Moreover, it has been demonstrated that in human arteries Ang II increases superoxide levels and that this effect, mediated by NOX, is inhibited by AT1 receptor antagonists (Berry et al., 2000). However, results with AT1 antagonists are more convincing in patients with coronary artery disease (Hornig et al., 2001) rather than with hypertensive subjects (Ghiadoni et al., 2000).

Finally, another interesting aspect of NOX involvement in blood pressure homeostasis impairment is related to cigarette smoke, a risk factor of hypertension. Cigarette smoke condensate exposures have been correlated with ROS production, downregulation of enzymatic antioxidant cellular systems and cell toxicity (Russo et al., 2011). NOX2 activation by cigarette smoke have been demonstrated in isolated blood vessels and cultured ECs and VSMCs, suggesting a role of NOX-derived ROS in endothelial dysfunction associated with hypertension (Kim et al., 2014).

## Homolog-specific NOXs inhibitors

NOXs are now being considered as target of pharmacological intervention in patients with hypertension (Cai et al., 2003). Homolog-specific NOX inhibitors have been recently developed. This class of drugs opens the possibility to affect ROS production without altering ROS beneficial effects. The peptide-based inhibitors like NOX2ds-TAT (Csányi et al., 2011) and the NOX1 targeting inhibitor, NOXA1ds (Ranayhossaini et al., 2013) are the most reliable in *in vitro* experiments. However, due to the low bio-availability, peptide inhibitors are not promising therapeutic tools.

A number of small molecule NOXs selective inhibitors have been developed. Among them there are NOX1 and NOX 4 selective inhibitors like GKT137831, GKT136901, and GKT901 (Takac et al., 2012). The first one is in a phase 2 trial in patients with diabetic nephropathy and is also subjected to a series of preclinical studies for its application in different disease including cardiovascular disease.

## Conclusions

**Figure 2:**
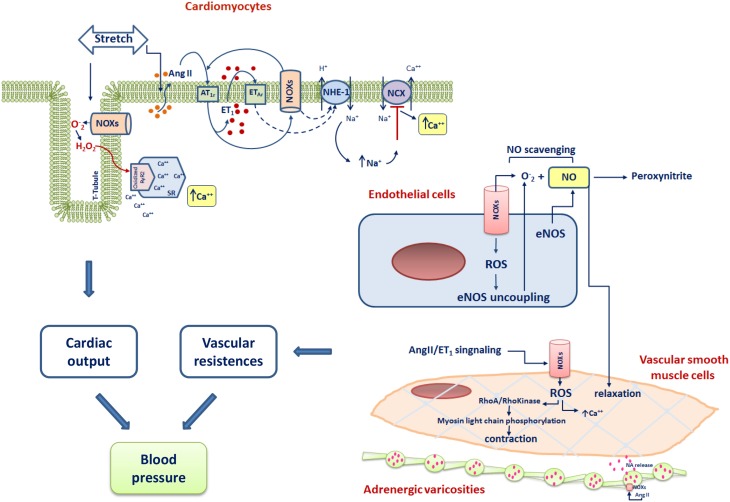
**Scheme of the main NOXs-dependent cardiovascular mechanisms involved in the control of blood pressure**. RyR2, Ryanodine Receptor type 2; ET-1, endothelin-1; NA, noradrenaline; AT_1_r, Angiotensin I type 1 receptor; ET_1_, endothelin 1; ET_*AR*_, Endothelin type A receptor; NHE-1, Na^+^/H^+^ exchanger-1; NCX, Na+/Ca2+ exchanger; e-NOS, endothelial nitric oxide synthase.

Multiple molecular mechanisms regulating blood pressure involve NOX signaling. Of great importance is the central role of NOXs in angiotensin signaling, in the availability of NO and in the cardiac mechanosensing. A general scheme of the main overall effects of NOX-mediated signaling in the cells of the cardiovascular system leading to blood pressure modulation are shown in Figure 2.

The current challenge in the NOX biology research field is represented by the better understanding of the mechanisms by which NOX isoforms exert their differential biological effects leading to the development of substances able to modulate specific redox-dependent cell functions.

## Conflict of interest statement

The authors declare that the research was conducted in the absence of any commercial or financial relationships that could be construed as a potential conflict of interest.
